# Deletion of a Yci1 Domain Protein of *Candida albicans* Allows Homothallic Mating in *MTL* Heterozygous Cells

**DOI:** 10.1128/mBio.00465-16

**Published:** 2016-04-26

**Authors:** Yuan Sun, Christine Gadoury, Matthew P. Hirakawa, Richard J. Bennett, Doreen Harcus, Anne Marcil, Malcolm Whiteway

**Affiliations:** a Biology Department, Concordia University, Montreal, Quebec, Canada; b Biotechnology Research Institute, National Research Council, Montreal, Quebec, Canada; c Department of Molecular Microbiology and Immunology, Brown University, Providence, Rhode Island, USA

## Abstract

It has been proposed that the ancestral fungus was mating competent and homothallic. However, many mating-competent fungi were initially classified as asexual because their mating capacity was hidden behind layers of regulation. For efficient *in vitro* mating, the essentially obligate diploid ascomycete pathogen *Candida albicans* has to change its mating type locus from heterozygous *MTL***a**/α to homozygous *MTL***a***/***a** or *MTL*α/α and then undergo an environmentally controlled epigenetic switch to the mating-competent opaque form. These requirements greatly reduce the potential for *C. albicans* mating. Deletion of the Yci1 domain gene *OFR1* bypasses the need for *C. albicans* cells to change the mating type locus from heterozygous to homozygous prior to switching to the opaque form and mating and allows homothallic mating of *MTL* heterozygous strains. This bypass is carbon source dependent and does not occur when cells are grown on glucose. Transcriptional profiling of *ofr1* mutant cells shows that in addition to regulating cell type and mating circuitry, Ofr1 is needed for proper regulation of histone and chitin biosynthesis gene expression. It appears that *OFR1* is a key regulator in *C. albicans* and functions in part to maintain the cryptic mating phenotype of the pathogen.

## IMPORTANCE

*Candida albicans* is a human fungal pathogen with a recently discovered, highly cryptic mating ability. For efficient mating, it has to lose heterozygosity at its mating type locus. Then, *MTL* homozygous strains can undergo an epigenetic switch to an elongated yeast state, termed the opaque form, and become mating competent. This infrequent two-step process greatly reduces the potential for mating; few strains are *MTL* homozygous, and the opaque state is unstable at the temperature of the mammalian host. *C. albicans* has a complex mechanism for mating that appears designed to ensure that mating is infrequent. Here, we have characterized a new gene, opaque-formation regulator 1 (*OFR1*). Deleting the *OFR1* gene allows *MTL***a**/α strains to mate efficiently with either mating type or even mate homothallically. It is possible that downregulating *OFR1* in the host environment could allow mating in *C. albicans* by a route that does not involve *MTL* homozygosis.

## INTRODUCTION

Most eukaryotes, from yeasts to humans, are sexual, and gametogenesis has evolved to increase diversity and improve species survival. Within the ascomycete fungi, the regulatory circuitries controlling mating are broadly similar, but the propensity for mating varies considerably. For fungi like *Saccharomyces cerevisiae*, heterothallic haploid cells are stable and mating is essentially constitutive for cells that are propagating vegetatively, so these cells couple mating with good environments (1, 2). For fungi such as *Schizosaccharomyces pombe*, mating is linked to nutrient limitation or other stressful conditions; these cells initiate mating in response to negative conditions in the environment (3). Other fungi such as *Candida albicans* successfully hid their sexuality until the genomic era (4); in these fungi, regulatory circuits demand very specific conditions for mating. Typically, *C. albicans* yeasts are diploid cells with the mating type **a**/α. These cells must first undergo loss of the heterozygosity at the *MTL* locus to become **a/a** and α/α and subsequently undergo an epigenetic switch to mating competency (5, 6). This limits *C. albicans* mating under laboratory conditions to specific genetic constitutions and environments; it is less clear what the situation is under natural conditions.

*C. albicans* represents the most prevalent opportunistic fungal pathogen colonizing humans. As a commensal yeast, it presents challenges for antifungal therapy due in part to its morphological diversity and flexibility (7). This flexibility includes the spontaneous and reversible cell morphological transition termed white-opaque switching, which is a prerequisite for gametogenesis and mating (8, 9). The white and opaque phases have differing properties in various aspects of the pathogen’s function, including its cellular morphology, staining with phloxine B, roles in systemic and cutaneous infections, adhesion, surface antigenicity, and specific gene expression profiles (9).

The mating type of *C. albicans* is controlled by the *MTL* (mating type-like) locus. This locus, located on chromosome 5, contains two alleles designated *MTL***a** and *MTLα* (10). Each locus contains a set of genes that include transcription regulators: **a**1 and **a**2 at *MTL***a** and α1 and α2 at *MTLα*. The α1 protein activates the α-specific genes, while **a**2 activates the **a**-specific genes (11). Furthermore, **a**1 and α2 combine to form a complex that represses the expression of various mating-related genes as well as white-to-opaque switching genes (12). Wor1 was identified as the key regulator of white-to-opaque switching controlled by **a**1-α2 repression. Deletion of *WOR1* blocks the transition to opaque cells in *MTL* homozygotes, whereas ectopic expression of *WOR1* can induce white-to-opaque switching in the *MTL***a**/α background. *WOR1* is in a bistable expression loop that is driven by feedback regulation; the positive feedback loop makes opaque cells stable after several cell divisions and the negative feedback loop makes the white-to-opaque transition easily reversible due to the influence of environmental factors (13–15).

Other potential regulators may participate in the feedback loops to strengthen the bistable expression of *WOR1*. Mating requires diploid **a**/α cells to make the *MTL* homozygous by either gene conversion or chromosome loss followed by duplication of the retained copy (9). This switching circuit is a unique step inserted before pheromone response in the mating process in *C. albicans* and its relatives *Candida dubliniensis* and *Candida tropicalis* (15–17). Even rare haploid strains selected through forced homozygosis of several loci still have to switch to the opaque phase to mate (18), and thus, the opaque state represents the “mating-competent” state. Only when environmental cues and conditions trigger the cells to enter the opaque phase can cells of the opposite sex then undergo mating. There is no similar switching required for mating among species of the *Saccharomyces* clade such as *S. cerevisiae*, *Kluyveromyces lactis*, and *Saccharomyces paradoxus*; these strains appear constitutively mating competent.

Various conditions have been identified that reduce the requirement for *MTL* homozygosity in the activation of the opaque state. Repression of the hemoglobin response gene *HBR1*, which is the activator of the mating type locus gene α2, allowed the mutant strains in the **a**/α background to undergo white-opaque switching and mating as **a** type cells (19). Recently, some clinical *MTL***a**/α isolates were identified that were capable of switching to opaque under specific conditions of 5% CO_2_ with *N-*acetylglucosamine (GlcNAc) as the carbon source. Deletion of the transcriptional factors Brg1, Rfg1, and Efg1, which are involved in repressing the positive feedback loop of *WOR1*, also allows white-to-opaque switching under 5% CO_2_-GlcNAc conditions (20). As well, specific conditions have identified alternative states, termed gray and gut cells. Gray cells were first discovered from a clinical isolate that when grown on yeast extract-peptone-dextrose (YPD) plates generated smooth gray colonies, distinguishable from white and opaque colonies (21). Gray cells were subsequently identified in different clinical isolates and shown to represent a third morphological type generated by a white-gray-opaque tristable switch controlled by Efg1 and Wor1. These gray cells have distinctive transcription profiles but have cell morphologies similar to the opaque haploids. As well, gut cells were found in genetically engineered strains that constitutively overexpressed *WOR1* after cell passage through the murine gut; these cells have a transcriptome compatible with the conditions found in the digestive tract but a morphology similar to opaque cells (22).

We have investigated further controlling elements of mating in *C. albicans*. Here, we identified white-opaque switching cells from a library of *MTL***a**/α strains that were mutant in nonconditional, nonessential genes and grown on GlcNAc agar medium. A strain defective in *orf19.5078* was identified as permitting efficient switching of *MTL***a**/α cells to the opaque state. *ORF19.5078*, which we have designated *OFR1* for opaque formation regulator, has not previously been characterized and has no ortholog in *S. cerevisiae*. By reconstructing the *ofr1* null mutant (*ofr1Δ/Δ*), we established that the complete deletion mutant also has the ability to bypass normal regulation and undergo white-opaque switching on GlcNAc medium when in the *MTL***a**/αstate. These opaque cells are fully mating competent, and thus *OFR1* functions, in an environmentally dependent manner, to maintain the cryptic mating state of *C. albicans*.

## RESULTS

### Screen for genes involved in white-opaque switching.

In the fungal pathogen *C. albicans*, the white-opaque transition is typically restricted to cells homozygous for the *MTL* locus, as the **a**1-α2 repressor prevents expression of the transcription factor *WOR1* required for formation of the opaque state (13–15). We screened for additional genes involved in white-opaque switching using a mutant library, GRACE version 1.0 (unpublished data), containing approximately 900 *MTL* heterozygous strains disrupted for nonessential genes to identify mutants that were capable of undergoing white-opaque switching when cells were cultured on GlcNAc medium at room temperature (RT). The entire library collection was suspended in 20% glycerol-supplemented YPD medium and stocked in 96-well microtiter plates. Stock plates were mixed on a microplate mixer and then robotically pinned to GlcNAc agar medium containing phloxine B to identify dark-staining opaque cell sectors. After a week’s incubation at room temperature, those colonies that formed pink (potentially opaque) sectors were restreaked and reincubated on fresh GlcNAc agar medium with phloxine B. Pink colonies were checked by optical microscopy to identify opaque-cell-like mutants, and heterozygosity at *MTL* was confirmed through colony PCR amplification of the *MTL***a**1 and -α2 genes.

**FIG 1:**
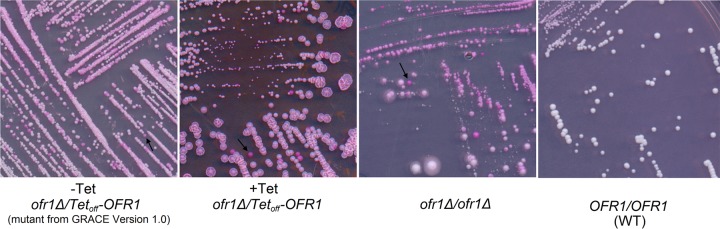
*ofr1* mutants undergo white-opaque switching on YCB-GlcNAc. Cells were streaked on YCB-GlcNAc agar plates containing phloxine B; under these conditions, opaque colonies are stained pink (representative colonies are noted with arrows). The mating types of all the strains are heterozygous *MTL***a***/α.* The *ofr1Δ/*Tet_off_*OFR1* (*MTL***a***/α*) strain is from the GRACE library and was streaked on a YCB-GlcNAc agar plate with 100 µg ml^−1^ tetracycline (*OFR1* expression is repressed by tetracycline). The *ofr1Δ/ofr1Δ* null mutant is derived from the wild-type strain SN148. *OFR1/OFR1* is SN148 as the control. These strains were all incubated at room temperature (RT) for 7 days before being scanned.

Further confirmation of a role in white-opaque switching was accomplished by testing the phenotype of the equivalent mutant from the original GRACE library. Because the GRACE library is a collection of mutant strains with one allele deleted and the other allele under Tet control (23), we examined the strains for white-opaque switching on 100-µg/ml-tetracycline-supplemented yeast carbon base (YCB)-GlcNAc-plus-phloxine B agar medium. For candidate genes, we then examined the phenotype of null mutants that were constructed in the SC5314 background. One of the strains identified, the *orf19.5078* strain, consistently showed enhanced white-opaque switching in the *MTL***a**/α background (Fig. 1). We designated this mutant gene *OFR1*, based on its resultant phenotype as an opaque formation regulator. *OFR1* has no clear ortholog in *Saccharomyces cerevisiae*, and the function of the encoded protein is currently undefined.

### Ofr1 has a conserved Yci1-related domain.

**FIG 2:**
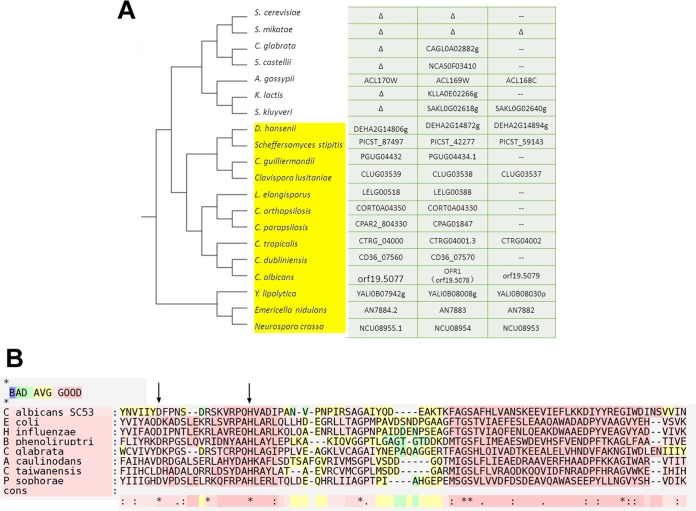
Bioinformatics analysis of *OFR1*. (A) Ortholog cluster of *OFR1* (*ORF19.5078*). There are orthologs of *OFR1* in most of the CTG clade of *Candida* species but no ortholog in *S. cerevisiae* and the close relatives of the budding yeast. The dendrogram on the left represents the phylogenetic tree of a selected set of ascomycetes. The genes shown on the right establish the syntenic arrangement of *OFR1* and its orthologs in these species. In many species, the genes flanking the *OFR1* orthologs are themselves orthologous, but in *S. cerevisiae* and close relatives, all three orthologous genes are missing or not syntenic in the genome. “Δ” indicates that the gene is absent from the genome, and “--” represents that the gene is elsewhere. (B) T-COFFEE protein analysis among several Yci1 domain proteins**.** The asterisk is used to label identical residues in the consensus sequence. The Yci1 domain has been proposed to have enzymatic function with putative active site residues Asp and His; the positions of these candidate active site residues are conserved in Ofr1 as D7 and H22, respectively, in the consensus (noted with arrows).

We examined the phylogenetic distribution of *OFR1* (*ORF19.5078*) orthologs within the ascomycetes. There are orthologs in most of the CTG clade of *Candida* species (Fig. 2A). There is also an ortholog in *Candida glabrata*, but none in *S. cerevisiae* and the close relatives of the budding yeast. Some species, such as *Scheffersomyces stipitis* and *Candida lusitaniae*, have 2 or 3 paralogs due to gene duplication.

We also investigated the domain architecture of Ofr1. The Ofr1 protein consists of 135 amino acids and contains a single Yci1-related domain located from amino acids R_41_ to P_114_. Yci1-related domains belong to a dimeric alpha-beta barrel protein family. This domain is widespread in eukaryotes, archaea, and a large group of both Gram-positive and -negative bacteria, and the structure of a family member from *Haemophilus influenzae* has been determined from protein crystals (24). The Yci1 domain has been proposed to have enzymatic function (one member of the family was identified as a dechlorinase [25]) with putative active site residues His and Asp; the position of these candidate active site residues is conserved in Ofr1 (Fig. 2B) (24). Overall, the role of the Yci1 domain is currently poorly defined, but a role in regulation of gene expression has been proposed because the domain is fused to sigma elements in *Streptomyces coelicolor* (26), and other family members are associated with operons that are connected to gene expression control (25).

### Ofr1p plays a carbon source-dependent role in white-opaque switching.

**FIG 3:**
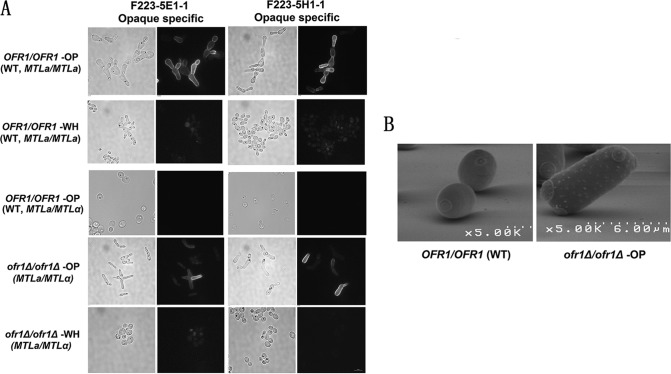
Microscopy. (A) Immunofluorescence microscopy of *ofr1* cells. Cells were fixed with formaldehyde and washed with 1× PBS. Fixed cells were stored in 1× PBS at 4°C before immunofluorescence microscopy. F223-5E1-1 and F223-5H1-1 are two different monoclonal antibodies used in this study as primary antibodies to identify opaque cells (see Fig. S1 in the supplemental material). Texas Red-conjugated goat anti-mouse antibody was used as the secondary antibody. OP, opaque cells; WH, white cells. Samples were observed and photographed under the Nikon Eclipse TiE fluorescence microscope at ×400 magnification. Bar, 10 µm. (B) Scanning electron microscopy. Cells were fixed in glutaraldehyde as described in the text after growth on YCB-GlcNAc agar plates at 25°C for 72 h. The samples were coated with 20 nm of gold palladium in an Emitech K550 sputter coater. Cells were photographed under a scanning electron microscope at ×5,000 magnification. *OFR1/OFR1* (WT) is the wild-type SN148 *MTL***a***/α* strain as the control. *ofr1Δ/ofr1Δ*-OP represents the opaque cell of the *ofr1* null mutant.

An *ofr1* null mutant strain constructed from the parent strain SN148 generated frequent pink (opaque) colonies when streaked on YCB-GlcNAc agar medium after a few days of cultivation at RT. Microscopic examination of the white colonies showed white-phase cells similar to the parent wild-type (WT) cells, while pink colonies showed elongated yeast cells with large vacuoles similar to opaque-phase cells. We performed a further analysis of the opaque-like phenotype by immunofluorescence microscopy, using two monoclonal antibodies that can differentiate white and opaque cells. When assayed using fluorescence microscopy, the opaque-like cells from the *MTL***a/**α *ofr1*Δ/Δ null mutant gave staining patterns similar to the classic opaque cells of the *MTL***a**/**a** wild type (Fig. 3A). The white cells from both the null mutant *MTL***a/**α and wild-type *MTL***a/a** strains showed no signal, as did the cells treated with only the secondary antibody.

Additionally, scanning electron microscopy (SEM) was used for analyzing the cell surface of the *ofr1* mutant. Gut cells were discovered from passage through the murine gut, and they expressed an optimized transcriptome for the digestive tract. They have opaque-like cell shapes but fail to respond to pheromone and showed no pimples under a scanning electron microscope (22). On the cell surface of the opaque-like cell formed by the mutant, there are the characteristic opaque pimples (Fig. 3B); this further supports the opaque status of the mutant cells.

Purified white and opaque colonies were selected to further test the overall white-opaque switching patterns. When individual cells from purified white colonies of the null mutant were incubated on YCB-GlcNAc agar medium at RT, the frequency of the opaque-like-form, phloxine-staining colonies was around 2%, while no switching of wild-type strain SN148 was observed under the same conditions (Table 1, rows 1 and 2). Purified opaque-like colonies treated in the same manner generated about 68% white/32% phloxine-staining opaque colonies (Table 1, row 11). Intriguingly, the switching rates of the *ofr1* mutant strains were carbon source dependent. Opaque *ofr1* cells from a GlcNAc plate would switch back quantitatively to white cell colonies when plated on YCB-glucose agar medium (Table 1, row 14), while white *ofr1* cells were unable to switch to opaque-form cells when cultured at room temperature on YCB-glucose agar medium (Table 1, row 6). Opaque-like cells formed by the *ofr1 MTL* homozygous strain were also quite unstable compared with wild-type (WT) *MTL* homozygous opaque cells under glucose growth conditions (Table 1, rows 16 and 17).

**TABLE 1:** Ratio of white-opaque switching^a^

Row	Strain			Carbon source		Switch ratio (%)
	Relevant genotype		Initial cell type	Starter colony	Scored cells	
	*ofr1*	*MTL*				
1	WT	**a**/α	WH	GlcNAc	GlcNAc	<0.05
2	Δ/Δ	**a**/α	WH	GlcNAc	GlcNAc	1.92
3	WT	**a/a**	WH	GlcNAc	GlcNAc	90.91
4	Δ/Δ	**a/a**	WH	GlcNAc	GlcNAc	94.78
5	WT	**a**/α	WH	GlcNAc	Glucose	<0.01
6	Δ/Δ	**a**/α	WH	GlcNAc	Glucose	<0.05
7	Δ/Δ	**a**/α	WH	GlcNAc	Glucose + CO_2_	<0.02
8	WT	**a/a**	WH	GlcNAc	Glucose	4.91
9	Δ/Δ	**a/a**	WH	GlcNAc	Glucose	3.93
11	Δ/Δ	**a**/α	OP	GlcNAc	GlcNAc	68.21
12	WT	**a/a**	OP	GlcNAc	GlcNAc	0.14
13	Δ/Δ	**a/a**	OP	GlcNAc	GlcNAc	<0.1
14	Δ/Δ	**a**/α	OP	GlcNAc	Glucose	99.6
15	Δ/Δ	**a**/α	OP	GlcNAc	Glucose + CO_2_	99.5
16	WT	**a/a**	OP	GlcNAc	Glucose	0.17
17	Δ/Δ	**a/a**	OP	GlcNAc	Glucose	98.05

a Carbon sources used were GlcNAc and glucose. Strains were either white (WH) or opaque (OP). The ratios are based on at least 2 separate experiments; colony types were calculated from among 200 to 1,000 colonies in total after 7 days of incubation at room temperature.

Another condition for inducing white-to-opaque switching in *C. albicans* is to incubate cells in 5% CO_2_. We tested 5% CO_2_ incubation but found that on YCB-glucose agar medium, white cells of the null mutant did not switch to opaque even after several days of incubation (Table 1, row 7), and opaque cells of the null mutant would all revert back to white cells after a 3-day incubation (Table 1, row 15). The *ofr1 MTL* heterozygous mutant could even form opaque colonies at mammalian body temperature, 37°C. At this temperature, in GlcNAc medium, the frequency of white-to-opaque switching was around 0.5% and the frequency of opaque-to-white switching was close to 90%.

### Opaque-phase stability of *ofr1* null mutants is carbon source dependent.

We tested the null mutant on other carbon sources to observe white-opaque switching. Galactose, mannitol, fructose, and xylose were all tested, together with glucose and GlcNAc as carbon source controls. Only GlcNAc could efficiently trigger the null mutant to switch to the opaque state. All *ofr1* mutant opaque cells were unstable and could revert to the white state, but this reversion was also carbon source dependent. Opaque cells transferred to glucose medium would switch efficiently to the white state, generating 100% white colonies, while reculturing to GlcNAc medium resulted in only ~70% white colonies (Table 1, rows 14 and 11, respectively).

### Mating ability of *ofr1* mutant.

**FIG 4:**
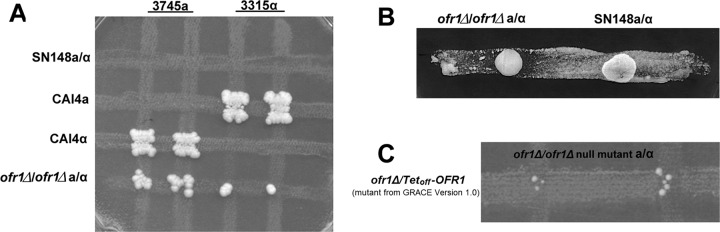
Mating ability of *ofr1* mutant. (A) **A**n *ofr1Δ/Δ* strain undergoes mating with both wild-type *MTL* homozygous strains. Strains 3745**a** and 3315α, in the opaque state, were used as mating type testers. They had the auxotrophic markers *trp1/trp1* and *lys2/lys2*. These testers were crossed with WT strains SN148 **a**/α (*arg4/arg4 leu2/leu2 his1/his1 ura3*::*imm434/ura3*::*imm434*), CAI4 **a** (*ura3*::*imm434/ura3*::*imm434*), and CAI4 α (*ura3*::*imm434/ura3*::*imm434*) and the *ofr1* **a***/α* null mutant (*arg4/arg4 leu2/leu2*) on GlcNAc medium at RT for 2 days and then replicated on selection medium YCB-glucose (Trp^−^ Lys^−^ Arg^−^ Ura^−^) at 30°C for 3 days to detect auxotrophic mating products. (B) Pheromone response assays. Approximately 5 × 10^6^ opaque cells of the highly pheromone-sensitive *cpp1Δ/Δ MTL***a***/***a** strain were evenly streaked onto YCB-GlcNAc agar medium. Spots of the SN148 **a**/α (wild type, white cells) and *ofr1* **a***/α* (*MTL***a***/α*, opaque cells) strains were assessed for pheromone production. SN148 **a**/α represents the negative control. Single colonies of *ofr1* **a***/α* and SN148 **a***/α* cells from GlcNAc agar medium were separately suspended in 20 µl MilliQ sterile water. Five microliters was used for spotting onto the hyperresponsive cell streaks, and the plate was incubated at 25°C for 48 h prior to scanning. (C) Homothallic mating of *ofr1*Δ/Δ cells***.*** Opaque cell colonies of the *ofr1* **a***/α* null mutant (*arg4/arg4 leu2/leu2*) and the *ofr1* **a***/α* GRACE1.0 (*ura3/ura3*) strains were mixed on GlcNAc medium for 2 days and then replicated on selection medium (Arg^−^ Ura^−^) to detect auxotrophic mating products.

We used wild-type tester strains 3315α (*MTLα/α*) and 3745**a** (*MTL***a/a**) to investigate the mating properties of the *MTL***a**/α *ofr1* mutant strain switched to the presumptive opaque state. We observed prototrophic colonies arising from auxotrophic marker complementation of both testers within 3 days of incubation on YCB-glucose selection medium after initial culturing on YCB-GlcNAc medium: the prototrophs were stable and represented true mating products. The mating assay thus suggested that the *MTL***a***/α ofr1* mutant could undergo mating with both *MTLα/α* wild-type cells and *MTL***a/a** wild-type cells when the *ofr1* opaque state was stabilized on YCB-GlcNAc medium (Fig. 4A; Table 2).

**TABLE 2:** Quantitative mating assays^a^

Tester strain	Type	*OFR1* genotype	Exptl strain	Type	*OFR1* genotype	Mating frequency
3315 α	OP	WT	SN148 **a**/α	WH	WT	<1.22 × 10^−10^
3315 α	OP	WT	SN148 **a/a**	OP	WT	(9.3 1 ± 5.69) × 10^−4^
3315 α	OP	WT	*ofr1* **a**/α	OP	*ofr1Δ/ofr1Δ*	(1.15 ± 0.03) × 10^−6^
3315 α	OP	WT	*ofr1 α*	OP	*ofr1Δ/ofr1Δ*	<1.02 × 10^−10^
3745 **a**	OP	WT	SN148 **a**/α	WH	WT	<5.78 × 10^−10^
3745 **a**	OP	WT	*ofr1* **a**/α	OP	*ofr1Δ/ofr1Δ*	(4.06 ± 0.53) × 10^−4^
3745 **a**	OP	WT	*ofr1 α*	OP	*ofr1Δ/ofr1Δ*	(1.21 ± 0.18) × 10^−2^
3745 **a**	OP	WT	*ofr1* **a**	OP	*ofr1Δ/ofr1Δ*	<1.44 × 10^−9^
*ofr1* **a**/α 1.0	OP	*ofr1*Δ/Tet_off_*OFR1*	*ofr1* **a**/α	OP	*ofr1Δ/ofr1Δ*	(9.80 ± 3.76) × 10^−7^
*ofr1 α* 1.0	OP	*ofr1*Δ/Tet_off_*OFR1*	*ofr1* **a**	OP	*ofr1Δ/ofr1Δ*	(1.27 ± 0.09) × 10^−2^

a Tester strains and experimental strains were precultured in YCB-GlcNAc liquid medium for 24 h and then mixed in fresh YCB-GlcNAc liquid medium at a concentration of 1 × 10^7^ cells/ml for both strains. Mixed cells were incubated at RT for 48 h and then plated onto selection medium to detect auxotrophic mating products. The mating frequency is calculated as described in Materials and Methods. OP, opaque; WH, white.

This mating is not the result of rare *MTL* homozygosity arising within the *MTL***a**/α *ofr1* population. We confirmed the presence of the **a**1 and α2 genes by colony PCR before performing the mating assays. As well, the mating frequency of the *MTL***a**/α *ofr1* mutant is around 4 × 10^−4^ with *MTL***a** cells and 1 × 10^−6^ with *MTLα* cells (Table 2); these values are close to the frequencies seen for mating of a control *MTL* homozygous strain (9 × 10^−4^ with the *MTLα* tester) and much higher than the undetected (less than 1 × 10^−10^) frequency of a wild-type *MTL* heterozygote with *MTL* homozygotes. We further investigated whether the frequency of *MTL* homozygosity was elevated in *ofr1* mutant cells. We continuously restreaked the null mutant onto GlcNAc agar medium and retested opaque colonies for the *MTL* loci by colony PCR. The heterozygosity of the *MTL* loci was stable during weeks of culturing. A direct assay for potential loss of heterozygosity at the *MTL* loci involves growth on sorbose medium; homozygosis of chromosome 5, which contains the *MTL* locus, allows growth on sorbose medium (27). We found the frequency of colonies identified after sorbose selection in the *ofr1* mutants to be low, in fact lower than the rate characteristic of wild-type **a/**α cells under sorbose selection (data not shown). Further, when we characterized the sorbose-resistant colonies from the *ofr1* mutant strain, we observed a high frequency of colonies that survived the sorbose selection without loss of heterozygosity (LOH) at the *MTL* locus. *ofr1* null mutant cells thus appear unusually stable for heterozygosity at the *MTL* locus. The somewhat-lower-than-wild-type mating efficiency even on GlcNAc medium may result from the switching of the *ofr1* mutant from opaque to white form, since *ofr1* opaque cells are quite unstable and we observed a 68% switching ratio on YCB-GlcNAc medium. This observation importantly suggests that *ofr1 MTL***a**/α mutants fully resemble *MTL* homozygous wild-type cells in that they also need to first switch to the opaque state to become mating competent.

We performed pheromone response assays to check if *MTL***a**/α *ofr1* cells can produce the α-factor pheromone. These assays showed that *MTL***a**/α *ofr1* opaque cells can cause sensitive *MTL***a** opaque cells to arrest similarly to the *MTLα* opaque cells (Fig. 4B) (28). *MTL***a**/α wild-type cells failed to cause *MTL***a** opaque cells to arrest. The cells in the zone of inhibition form shmoos (see Fig. S2A in the supplemental material). Pheromone response assays were also performed to confirm that *MTL***a**/α *ofr1* mutants can respond to α pheromone, and we detected shmoo tubes after 24-h pheromone treatment in liquid medium (see Fig. S2B).

Since *MTL***a/**α *ofr1* opaque cells can undergo mating with both types of *MTL* homozygous wild-type opaque cells, we assessed if deletion of *OFR1* could allow *MTL***a**/α opaque cells to mate among themselves. We performed a mating assay between the *MTL***a**/α *ofr1* null mutant and the *MTL***a**/α *ofr1* mutant from the GRACE 1.0 library; these strains had complementing auxotrophies. We observed prototrophic colonies arising from auxotrophic marker complementation within 3 days of incubation on YCB-glucose selection medium after initial culturing on YCB-GlcNAc medium (Fig. 4C). We assayed the prototrophic mating products arising through mating among these *ofr1 MTL***a**/α mutants for DNA content and found them to be the expected tetraploids with twice the content of the diploid parents (data not shown). As well, strains with *MTL***a**1 polymorphisms were used for mating, and sequencing the prototrophic mating products showed a combination of the two allelic polymorphisms (see Fig. S3 in the supplemental material). The quantitative mating assay showed a mating frequency of around 1 × 10^−6^ between the *MTL***a**/α *ofr1* null mutant and the *MTL***a**/α *ofr1* mutant from the GRACE 1.0 library (Table 2).

### Comparison of transcription profiles between *ofr1* mutant and wild-type white cells on glucose-containing medium.

*OFR1* expression is regulated by neither white-opaque switching nor pheromones according to compilations of transcription data provided by the CGD database. We used transcription profiling to examine the differences in gene expression levels between the *ofr1* null mutant and the wild type under normal glucose growth conditions. Since the *MTL***a**/α wild type cannot form opaque cells, we used microarrays to compare the *ofr1* **a**/α white cells with wild-type **a**/α white cells for gene expression under glucose incubation conditions at room temperature after 12 h. Under the glucose conditions, hundreds of genes were significantly upregulated and a few genes were significantly downregulated in the *ofr1* null strains (see Table S1 and Fig. S4A and B in the supplemental material). Among these modified genes were *WOR1* (upregulated) and *WH11* (downregulated) in *ofr1* **a***/α* white cells (Table 3)*.* As well, mating pheromone precursor genes *MFA1* and *MFα1* were both upregulated in *ofr1* **a**/α cells, as were both **a** and α pheromone receptor genes *STE3* and *STE2.* The G protein subunit genes *CAG1* and *STE18* were upregulated along with other genes involved in mating and pheromone response, such as *HST6*, *IFA4*, *AKL1*, *PRM1*, *FUS1*, and *CTF5*. Genes involved in carbohydrate transport and utilization were also upregulated in the null mutant background (Table 3). Under these growth conditions, 8 out of 19 glucose transporter family members (*HGT1*, *HGT12*, *HGT13*, *HGT17*, *HGT2*, *HGT6*, *HGT9*, and *HXK2*) were upregulated in the mutant relative to the wild type; chitin synthesis genes *CHS1*, *CHS2*, and *CHT3* as well as other genes involved in carbohydrate metabolism, including *CIT1*, *CRH11*, *PGM2*, *PHR3*, and *SCW11*, are also more highly expressed in the mutant. Similarly, *SAP* and *LIP* genes, which affect the maturation of pheromones and phenotype switching, were also upregulated, including *SAP1*, *SAP30*, *SAP8*, *SAP2*, *SAP5*, *SAP7*, *LIP4*, *LIP2*, and *LIP1*. Overall, these expression characteristics highly resembled the gene expression profiles of white wild-type *MTL* homozygous cells treated with pheromone (29).

**TABLE 3:** Highlighted significant genes in white cells of the *ofr1* null mutant compared with white cells of the wild-type reference strain^a^

Category	Genes significant on:	
	GlcNAc	Glucose
White-opaque switching		*WOR1*, WH11*
Involved in mating and pheromone response	*PCL1*, *FAV3*, *EMC9*, *MET28*	*MNN4-4*, *orf19.5896*, *RBT4*, *SLF1*, *STE2*, *STE3*, *HST6*, *IFA14*, *orf19.104 MFALPHA*, *AKL1*, *PRM1*, *FUS1*, *MFA1*, *CTF5*, *CAG1*, *STE18*, *SAP30*
Other significant groups		
Histone genes	*HHF1*, *HHF22*, *HHT1*, *HHT2*, *HHT21*, *HTA1*, *HTA2*, *HTA3*, *HTB1*, *HTB2*	
Chitin synthesis genes	*CHS1*, *CHT3*	*CHS1*, *CHS2*, *CHT3*
Alcohol dehydrogenase	*ADH1*, *ADH2*, *FDH3*	*ADH3*
SAPs and LIPs		*SAP1*, *SAP30*, *SAP8*, *SAP2*, *SAP5*, *SAP7*, *LIP4*, *LIP2*, *LIP1*
Glucose transport	*HGT6*, *HGT13*, *HGT17*	*HGT1*, *HGT2*, *HGT6*, *HGT9*, *HGT12*, *HGT13*, *HGT17*, *HXK2*
Carbohydrate metabolism	*ADH1*, *ADH2*, *ALG8*, *AMS1*, *ARA1*, *BMH1*, *CDC19*, *CHS1*, *DAK2*, *GLK4*, *IPP1*, *KAR2*, *MDH1*, *MNT1*, *PDA1*, *PFK2*, *PGM2*, *RHO1*, *SCW11*, *SRB1*, *UGP1*, *ZWF1*	*CIT1*, *CRH11*, *PGM2*, *PHR3*, *SCW11*, *GAL1*, *GUP1*, *MAL31*

a Highlighted significant genes changed in expression for the **a**/α *ofr1* null mutant in the white state compared with the wild-type **a**/α SN148 strain under both GlcNAc and glucose conditions. Microarrays were based on at least two replicates with dye swaps. Log_2_, >1 or <−1; *P* < 0.1. A downregulated gene is noted with an asterisk. For details, see Table S1 and Fig. S2 in the supplemental material.

### Comparison of transcription profiles between *ofr1* mutant and wild-type white cells on GlcNAc medium.

Since *ofr1* white cells switch to the opaque form opaque specifically on GlcNAc medium, we also investigated white mutant and wild-type cells using transcription profiling on this carbon source. We compared the transcriptional profile of the **a**/α *ofr1* white cells (which have the potential to switch to the opaque state) with that of the **a**/α wild-type white cells (which have no potential to switch to opaque) to see if *WOR1* expression was misregulated in the *ofr1* mutant cells grown on GlcNAc. This analysis revealed that *WOR1* gene expression, as well as that of other white-opaque switching-related transcriptional factors and genes, was not dramatically changed in the null mutant *ofr1* white cells in GlcNAc compared with wild-type **a**/α SN148 white cells after a 12-h incubation at RT. The *ofr1* strain exhibited general differences in genes implicated in processes such as carbohydrate metabolism, oxidation/reduction, ATP metabolism, nucleosome organization, and RNA processing (see Fig. S4C and D in the supplemental material). Under GlcNAc growth conditions, the *ofr1* mutant did not significantly modulate transcripts related to the Wor1 circuit but did upregulate transcripts for genes involved in glycolysis, such as *PFK2*, *TPI1*, *TDH3*, and *CDC19*; genes involved in fermentation, such as *ADH1*, *ADH2*, *ADH5*, and *ORF19.3045*; and genes involved in UDP-glucose conversion, such as *PGM2*, *IPP1*, and *UGP1*. Intriguingly, histone genes are dramatically upregulated in the mutant when grown on GlcNAc. All 10 histone genes (*HTA1*, *HTA2*, *HTA3*, *HTB1*, *HTB2*, *HHT1*, *HHT2*, *HHT21*, *HHF1*, and *HHF22*) were among the top upregulated genes (7 of 10 showed values >2-fold of the log_2_ ratio [top 30] between the mutant and the wild type) (Table 3). Together, these histone genes encode a histone octamer composed of two histone H2A-H2B dimers and one histone tetramer (H3-H4)_2_. It has been reported that posttranslational modifications of histones or chromatin can influence the white-opaque transition (30). Based on these microarray data, it is possible that the potential of the white, **a**/α *ofr1* mutant strains to switch to the opaque state is a consequence of this improper histone gene expression.

### Comparison of transcription profiles between the *ofr1* a/α opaque and white states.

We wanted to establish if the transcription profile difference between the *ofr1* **a**/α opaque and white cells was similar to that of the wild-type **a/a** opaque cells compared with white cells. Because *ofr1* opaque cells were relatively stable when incubated in liquid YCB-glucose medium at RT for 12 h, and the published white/opaque data sets were derived from glucose-grown cells, we transferred both white and opaque cells from YCB-GlcNAc medium and grew them separately in liquid YCB-glucose medium for 12 h before collecting the cells for RNA extraction. We investigated the *ofr1* **a**/α opaque cells compared with white cells for gene expression under the glucose incubation conditions, and we compared these microarray data with those of the *MTL***a/a** wild-type opaque/white comparison from the literature (31, 32). The genes significantly up- and downregulated suggest that the *ofr1* opaque cells follow the expression patterns of classic opaque cells for key functions (see Fig. S5 in the supplemental material). The *MTL***a**/α *ofr1* opaque cells cultured on glucose turn up the Wor1 circuit upregulated genes (*WOR1*, *WOR2*, and *CZF1*); they turn down typically downregulated genes (*EFG1* and *WH11*) (Table 4). They also upregulate Krebs cycle genes (*PYC2*, *PDC11*, *IDP2*, *SDH1*, *SDH2*, *FAA4*, and *CIT1*), as previously noted for standard opaque cells (Table 4) (32). In addition, the *MTL***a**/α *ofr1* opaque cells upregulate genes involved in carbohydrate transport (*ORF19.4923*, *ORF19.1867*, *ORF19.3782*, *MNT4*, and *MNN11*) and utilization (*ORF19.3325*, *HGT8*, *GAL10*, *GAL1*, *ORF19.1340*, *UTR2*, *FBP1*, *ORF19.2308*, *GCA2*, and *GUT2*) relative to the white cells. The GlcNAc metabolism genes (*HXK1*, *NGT1*, *GFA1*, *DAC1*, and *NAG1*) are also dramatically upregulated, which is potentially a residue of the transition from the initial GlcNAc growth conditions. Interestingly, we have histone genes (*HHF1*, *HHF22*, *HHT21*, *HTA2*, and *HTB1*) and chitin synthesis genes (*CHS1*, *CHS8*, *CHS5*, and *CHS7*) highly expressed in the *ofr1* **a**/α opaque cells relative to the white cells; these genes were not upregulated in the classic opaque cells.

**TABLE 4:** Highlighted significant genes in opaque cells compared with white cells of the *ofr1* null mutant^a^

Category	Genes significant on:	
	GlcNAc	Glucose
White-opaque switching	*WOR1*	*WOR1*, *WOR2*, *CZF1*, EFG1*, WH11*
Krebs cycle genes		*PYC2*, *PDC11*, *IDP2*, *SDH1*, *SDH2*, *FAA4*, *CIT1*
Involved in mating and pheromone response	*CEK2*, *RMS1*, *RSN1*, *PRM1*, *SAP30*	*CEK2*
Other significant groups		
Histone genes		*HHF1*, *HTB2*, *HHF22*, *HTA2*, *HTB1*, *HHT21*
Chitin synthase		*CHS1*, *CHS8*, *CHS5*, *CHS7*, *GFA1*
SAPs and LIPs	*SAP30*, *SAP4*, *SAP98*, *LIP7*	*SAP10*
ALS family protein	*ALS2*, *ALS4*, *ALS9*	*ALS3*
Glucose transport	*HGT1*, *HGT6*, *HGT7*, *HGT19*	*HGT8*
Carbohydrate metabolism	*ORF19.4923*, *AMS1*, *ATC1*, *BMT4*, *GAC1*, *GCA1*, *GPD2*, *GPM1*, *HSP104*, *INO1*, *KTR2*, *MAL2*, *MNN22*, *PFK26*	*ORF19.4923*, *ORF19.1867*, *ORF19.3782*, *MNT4*, *MNN11*, *ORF19.3325*, *GAL10*, *GAL1*, *ORF19.1340*, *UTR2*, *FBP1*, *ORF19.2308*, *GCA2*, *GUT2*
GlcNAc utilization		*DAC1*, *NAG1*, *HXK1*, *NGT1*, *GFA1*

a Highlighted significant genes changed in the gene expressions of the *ofr1* null mutant in opaque states compared with those in white states under both GlcNAc and glucose conditions. Microarray, based on at least two replicates with dye swaps. Log_2_, >1 or <−1; *P* < 0.1. Downregulated genes are noted with an asterisk. For details, see Table S2 and Fig. S4 in the supplemental material.

### Comparison of transcription profiles between the *ofr1* a/α opaque and white states on GlcNAc medium.

Finally, we also compared the gene expression between *ofr1* **a**/α opaque and white cells under the GlcNAc growth condition that allows the mutant cells to mate. Under these conditions, *WOR1* is upregulated in the opaque cells, and there were some significantly expressed genes that also showed up during glucose growth of the mutant (Table 4). Other upregulated genes in GlcNAc included genes involved in secretion, such as *SAP30*, *SAP4*, *SAP98*, and *LIP7*; in adhesion, such as *ALS2*, *ALS4*, and *ALS9*; and in glucose transport, such as *HGT1*, *HGT6*, *HGT7*, and *HGT19* (Table 4). Histone gene expression was not significantly changed here because all the histone genes were highly expressed in both white and opaque states on GlcNAc, but the expression levels were marginally higher in white cells.

## DISCUSSION

*Candida albicans* has a complex signaling pathway to regulate mating, involving receptor proteins, a heterotrimeric G protein, a scaffolded mitogen-activated protein (MAP) kinase cascade, and a transcriptional control module (33). However, the ability of this circuitry to trigger mating is maintained behind several layers of regulation. Most *C. albicans* cells are diploid and heterozygous at the mating type locus and thus blocked in mating because they cannot enter the opaque stage necessary for conjugation (34). Even mating type homozygous strains have to undergo an infrequent epigenetic switch to attain this mating-competent opaque state, a switch that is inherently difficult at 37°C, the temperature of the mammalian host (35). This situation is in contrast to many other ascomycetes with similarly structured mating regulation pathways, such as *S. cerevisiae* and *S. pombe*; these yeasts lack the epigenetic circuit, although they can link environmental signals to the mating decision.

We have identified that the *OFR1* gene, encoding a Yci1 domain protein, plays a key role in keeping the mating capacity of *C. albicans* cells cryptic. When this gene is deleted, it is possible for mating type heterozygous *C. albicans* cells to enter the opaque state and mate. This process is environmentally controlled, occurring when cells are grown on GlcNAc but not on other carbon sources such as glucose or galactose. This is consistent with the role of GlcNAc as an efficient inducer of opaque cell formation (36). This mating ability of *ofr1 MTL***a**/α cells is intriguing, because normally mating is precluded in cells heterozygous at the *MTL* locus. The *ofr1 MTL***a**/α cells are able to mate with *MTL***a** and *MTLα* cells and, to a lesser extent, with *ofr1 MTL***a**/αcells. The latter situation represents homothallic mating of *MTL***a**/α cells. Pheromone response arrest assays show that the *ofr1 MTL***a**/α cells can produce abundant α-factor, and pheromone response experiments show that the *ofr1 MTL***a**/α cells can also respond to α-factor and shmoo.

We used transcriptional profiling to probe gene expression in *ofr1* mutant strains. *MFA1* (*ORF19.2164.1*), the gene encoding the **a**-factor mating pheromone precursor, and *MF*α*1*(*ORF19.4481*), the gene encoding the α-factor mating pheromone precursor, are somewhat upregulated in the *ofr1 MTL***a**/α background compared with the wild type. Unisexual mating can happen in *C. albicans* through inhibition of Bar1 protease, which promotes autocrine signaling, or through the presence of α cells, which provide αpheromone (37). Addition of synthetic α pheromone or presence of αwhite cells is also sufficient to drive unisexual **a-a** mating in *C. albicans* (38). Both **a** and α pheromone receptor genes *STE3* and *STE2* are upregulated in the *ofr1* mutant, potentially allowing these cells to activate the mating response in the presence of either pheromone. By producing both the pheromones and the pheromone receptors, *ofr1 MTL***a**/α cells can undergo mating with either mating type. The previous reported *MTL***a**/α opaque cells are nonmating, probably because they cannot produce pheromones to induce mating. It appears that once these *C. albicans* cells enter the mating-competent opaque state, whether the mating type is *MTL***a**, *MTLα*, or *MTL***a***/α*, cells can undergo mating if presented with either **a** or α pheromones.

The overall transcriptional consequences of deletion of *OFR1* are influenced by the carbon source of the cell and are not limited to modulating genes involved in mating. Genes involved in carbohydrate metabolism, chitin synthesis, and histone production are all changed in the absence of *OFR1*, but these influences are also affected by whether the cells are grown on GlcNAc or glucose; in general, the transcriptional effects are greater for the cells grown on GlcNAc. These consequences in transcriptional regulation are intriguing given that *OFR1* is a member of the Yci1 family of proteins that have primarily been implicated in simple chemical reactions such as dechlorination (25). A family member was crystalized in *Haemophilus influenzae*; the protein formed an α/β ferredoxin-like fold and was complexed with ZnCl_3_ (24). This structure predicts a conserved His (H)-Asp (D) catalytic dyad, with Arg and Ser residues forming an oxyanion hole stabilized by a conserved Asp (24, 25). However, links to transcriptional control have been noted with several family members; in *Streptomyces coelicolor*, a Yci1 domain is fused to a sigma factor involved in global gene regulation (26), while in other bacterial species operons containing Yci1 family members are implicated in gene expression control and regulation of morphogenesis (25).

In *C. albicans*, it is possible that Ofr1 could serve directly or indirectly to strengthen the **a**1-α2 repression of *MTL* homozygous-specific gene expression. Without Ofr1p, the **a**1-α2 repressor complex could allow a low-level expression of *MTL*-repressed genes such as white-opaque switching-specific genes and pheromone response genes. If the threshold of switching to opaque on glucose is higher than that on GlcNAc, and deleting *OFR1* allows genes that are repressed by **a**1-α2 to be expressed at a relatively higher level than within the wild type, this could allow the mutant to pass the threshold of switching to opaque when grown on GlcNAc but not on glucose. As well, switching to opaque on GlcNAc might not be under the control only of the Wor1 circuit; there may be other potential regulators when the cells are grown on GlcNAc. This explanation is also suggested by the fact that the opaque cells formed by the *ofr1* null mutant on GlcNAc would all switch back to white cells on glucose. If GlcNAc turns on the Wor1 circuit, the effects should keep going under glucose conditions. This instability of the **a**/α opaque cells formed on GlcNAc suggests other possible routes present under GlcNAc conditions to regulate the white-opaque switching. Histone gene dosage can affect histone modifications as well as gene transcription (39, 40). The highly expressed histone genes of the *ofr1* mutant may influence white-to-opaque switching on GlcNAc medium and affect white-opaque switching in both directions. It is also possible that Ofr1 involvement in the white-opaque switching process results from a role in chromatin assembly or structures that control the switch. Some opaque-specific genes were not upregulated in the *ofr1* opaque cells compared with control white cells. This might be caused by the instability of the opaque cells and the presence of α pheromone. It has been reported that αpheromone downregulates expression of some opaque-phase-specific genes (such as *OP4*) (41).

It has been noted that only 3 to 9% of clinical *C. albicans* isolates in nature are *MTL* homozygous (42, 43). If mating of *C. albicans* is limited to *MTL* homozygous cells, it should be a rare event in nature, particularly when coupled to a requirement for switching to the mating-competent opaque state. GlcNAc and other environmental factors may trigger white-to-opaque switching in *MTL***a/**α cells by upregulating the Wor1 circuit or modulating chromatin assembly. In certain niches in the host, Ofr1 activity could be repressed, which could allow *MTL* heterozygous cells to switch to opaque, produce pheromones, and undergo mating. Also, in the presence of *MTL* homozygous cells that are producing either **a** or α pheromone, *MTL* heterozygous opaque cells can become as mating competent as *MTL* homozygous opaque cells.

Overall, our study describes a single gene, *OFR1*, which influences the control of the barriers to mating in *Candida albicans*. The Ofr1 protein is needed to ensure that white-opaque switching, pheromone production, and mating are blocked in *MTL* heterozygotes, and it also regulates the expression of all histone genes in a carbon source-dependent manner. This suggests a critical role of the conserved Yci1 domain, which until now has been identified with simple chemical reactions but is implicated in more complex regulatory roles (24). As more than 90% of *C. albicans* clinical isolates are *MTL* heterozygous, the presence of a complex, but apparently restricted, mating pathway is intriguing. The evidence that single gene mutations can allow carbon source-dependent mating behavior of *MTL* heterozygous strains suggests the possibility that *in vivo*, *C. albicans* may exploit alternate routes to mating and that the apparently cryptic pathway may not be as hidden as it appears.

## MATERIALS AND METHODS

### Strains, media, and culture conditions.

A collection of 887 nonconditional strains inactivated for nonessential genes, termed the GRACE version 1.0 library (unpublished data), was derived from the GRACE library of regulated disruptions (23) by identifying, through growth on 5-fluoroorotic acid (5-FOA) medium (44), derivatives that had lost the transactivator cassette. This library was used to identify colonies that could undergo white-to-opaque switching in the **a**/α background. The entire library collection was suspended in 20% glycerol-supplemented YPD medium and stocked in 96-well microtiter plates. Fully defrosted stock plates were mixed well on a microplate mixer and then robotically pinned to rectangular plates of GlcNAc agar medium containing phloxine B to identify dark-staining opaque cell sectors. The original GRACE library versions of candidates were then tested with and without tetracycline to confirm any mutants that triggered inappropriate white-to-opaque switching. Subsequently, *C. albicans* strain SN148 was used as the parent strain to construct the complete *ofr1*Δ/Δ null mutant strain (see Table S3 in the supplemental material).

For cultivation, YCB media with glucose (2%) or GlcNAc (1.25%) were used. Plate cultures were grown at a density of 40 to 120 colonies per 90-mm plate. Other carbon sources, including galactose, fructose, and mannitol, were used at 2%. For opaque colony identification, phloxine B (5 µg ml^−1^) was added to the agar medium. For routine liquid cultivation, YPD (1% yeast extract, 2% peptone, and 2% glucose) was used.

### Strain construction.

*OFR1* was deleted by standard two-step disruptions using PCR products (45). The markers *HIS1* and *URA3* were amplified by PCR from plasmids pFA-CaHIS1 and pFA-CaURA3 using primers that provided homology to the flanking regions of *OFR1*. The *HIS1* and *URA3* markers were sequentially transformed to the parent strain SN148, and transformants were selected on SD-His^−^ and SD-Ura^−^ agar plates. Successful transformants were further confirmed by PCR. One pair of long oligonucleotides for deletion and three pairs of short oligonucleotides for confirmation were used for the PCRs (see Table S4 in the supplemental material).

### Phenotype switching.

White and opaque cells were all selected from single colonies on YCB-GlcNAc medium after 5 days at room temperature. Cells then were suspended in water, the cell concentration was adjusted, and the suspensions were plated on agar media containing 5 µg ml^−1^ phloxine B and different carbon sources. Plates were incubated at either 24°C or 25°C with 5% CO_2_. Data were collected and plates were scanned on the 7th day, and the frequency of sectored colonies was calculated by standard statistical methods.

### Microarrays.

The wild-type SN148 and the *ofr1*Δ/Δ null mutant were selected from single colonies and grown in either YCB-glucose or YCB-GlcNAc liquid medium overnight at RT and then were diluted to an optical density at 600 nm (OD_600_) of 0.1 in fresh YCB-glucose or YCB-GlcNAc liquid medium. Cells were grown at RT until the culture reached an OD_600_ between 0.8 and 1.2, harvested, and stored at −80°C until RNA extraction. Total RNA was isolated by the hot phenol method as described elsewhere (45). mRNA was purified using the New England Biolabs polyA Spin mRNA isolation kit, and then reverse transcription for cDNA production was followed by indirect cDNA labeling with aminoallyl-dUTP for dye addition. Arrays were obtained from the NRC-BRI Microarray Facility; hybridization protocols were as described previously (45).

### Microscopy and imaging.

Optical microscopic images of cells were captured using a Nikon Eclipse TS100 microscope. Immunofluorescence microscopic images were visualized and photographed using Nikon Eclipse TiE with ×400 magnification with the following settings: objective, 100× oil; filter, TxRed-560/40; dichroic beam splitter, bs585; emission, 630/75; excitation wavelength, 555 nm, using a Multilaser Heliophor and a Photometrics Evolve camera. Images of plates and colonies were scanned at 800 dots per inch (dpi) by an Epson Perfection v500 photo scanner.

### Custom monoclonal antibody generation reagents.

Iscove’s modification of Dulbecco’s medium (IMDM) supplemented with GlutaMAX-1 was purchased from Gibco (Life Technologies, Grand Island, NY, USA) and Dulbecco’s phosphate-buffered saline (D-PBS) was purchased from HyClone (Thermo Scientific, QC, Canada). Mouse interleukin-6 (IL-6) was purchased from Invitrogen (Burlington, ON, Canada). Commercial antibodies were purchased from Jackson ImmunoResearch (West Grove, PA). Unless otherwise cited, all other reagents were purchased from Sigma (St. Louis, MO). Fetal bovine serum (FBS) was heat inactivated at 56°C for 30 min.

### Antigen preparation and immunization.

A single opaque colony of *C. albicans* strain WO1 was grown in SD broth at 24°C, 200 rpm, for 24 to 30 h. The cells were harvested by centrifugation, washed twice in PBS, and resuspended to a density of 1.1 × 10^8^ cells/ml in PBS. One milliliter of cells was transferred to each well of a flat-bottom 6-well plate as a thin liquid suspension. Plates (without lids) were placed in a UV-DNA cross linker (CL-1000 from Ultra Violet Products [UVP]), and 7 doses of UV radiation (each 100 mJ/cm^2^ [254 nm]) at energy level 1200 were applied (≈30 s/dose). Plates were shaken gently between doses. Cells were scraped from each well and pooled for centrifugation, washed, and resuspended in PBS to a density of 1 × 10^8^ cells/ml. One-milliliter aliquots were centrifuged, supernatants were removed, and pellets were frozen at −80°C until resuspension for immunization. Before freezing, 5 × 10^6^ cells were plated on YPD solid medium and grown at 30°C to confirm killing.

Six-week-old female SJL mice (The Jackson Laboratory, Bar Harbor, ME) were immunized intraperitoneally and subcutaneously with 5 × 10^7^ cells of UV-killed *C. albicans* opaque strain WO1 emulsified in incomplete Freund adjuvant and boosted at days 21, 49, and 77 with the killed *Candida* cells diluted in PBS. A final boost was done at day 97 with 2 × 10^7^ UV-killed *Candida* cells 4 days prior to fusion.

### Hybridoma generation.

Splenocytes were fused to NS0 myeloma cell line cells (a kind gift from C. Milstein) at a ratio of 1:1 using an electrofusion apparatus (ECM 2001, BTX; Harvard Apparatus, Holliston, MA) according to the manufacturer’s instructions. After an overnight incubation in selection medium (IMDM supplemented with 20% FBS, 1× hypoxanthine-aminopterin-thymidine [HAT] supplement, 1 ng/ml mouse IL-6, 100 IU/ml penicillin, and 100 µg/ml streptomycin), freshly made hybridomas were washed and diluted in 90 ml of ClonaCell-HY hybridoma selection medium D (StemCell Technologies, Vancouver, BC, Canada) supplemented with 5% FBS, 1 ng/ml mouse IL-6, and 10 µg/ml of a fluorescein isothiocyanate (FITC)-labeled F(ab′)_2_ goat anti-mouse IgG. The cell suspension was plated in Nunc OmniTrays (Thermo Scientific) and incubated for 6 days at 37°C, 5% CO_2_.

### Clone picking and hybridoma screening.

A robotized fluorescent mammalian cell clone picker (ClonepixFL; Molecular Devices, Boston, MA) was used to pick secreting hybridoma clones. Picked colonies were transferred in 96-well plates containing 200 µl of the selection medium, except that HAT supplement was replaced with hypoxanthine-thymidine (HT) supplement, and picked clones were further incubated for 3 days at 37°C, 5% CO_2_. Hybridoma supernatants were screened for appropriate antigen specificity by cell enzyme-linked immunosorbent assay (cell-ELISA) on UV-killed opaque *Candida*. Briefly, 50 µl of a *Candida* opaque cell suspension at 2 × 10^7^ cells/ml in PBS containing 1% bovine serum albumin (BSA) (blocking buffer) was distributed in a 96-well MultiScreen-HTS filter plate (Millipore, Billerica, MA), incubated for 30 min at room temperature, and vacuum filtered; 50 µl of monoclonal antibody supernatant was applied; and microplates were shaken for 30 s. After a 90-min incubation at 37°C, 5% CO_2_, microplates were washed twice with PBS and 50 µl of a 1/4,000 dilution of alkaline phosphatase-conjugated goat anti-mouse IgG in blocking buffer was added. After a 1-h incubation at 37°C, microplates were washed 5 times and 50 µl of *p*-nitrophenyl phosphate (pNPP) substrate at 1 mg/ml in carbonate buffer at pH 9.6 was added and further incubated for 30 to 60 min at 37°C. Substrate was transferred to a transparent 96-well plate by vacuum, and absorbance was read at 405 nm using a SpectraMax plate reader (Molecular Devices, Sunnyvale, CA). Opaque specificity of the supernatants was confirmed by cell-ELISA testing on UV-killed hyphal and yeast forms of *Candida*. Confirmation on live *Candida* cells was done by immunofluorescence.

### Immunofluorescence.

Cells from single colonies cultured for 5 days on SD agar medium at RT were pregrown in SD liquid media overnight at RT, with 220-rpm shaking, and then diluted in fresh SD liquid medium for another 12-h incubation at RT, 220 rpm. Cells were fixed with a 1/10 volume of 37% formaldehyde (Fisher Scientific) in 1× PBS for 45 min at RT. The cells were pelleted and washed in 1 ml of 1× PBS twice for 45 min and stored in PBS at 4°C. The pelleted cells were washed in 1 ml of 1× PBS 3 times for 2 min each time; approximately 10^7^ cells were tested for each assay. Washed cells were blocked with 1 ml blocking buffer for 30 min before incubation with 100 µl of primary antibodies for another hour at RT. Cells were then washed with PBS containing 0.05% Tween 20 three times for 5 min each time. Washed cells were incubated with 100 µl of the secondary antibody, Texas Red-conjugated goat anti-mouse antibody (1/100 dilution in blocking buffer), for 1 h in the dark at RT. Cells were then washed with PBS containing 0.05% Tween 20 three times and PBS once, for 5 min each time. Cells were finally suspended in 50 µl PBS, and 3 µl was applied under a coverslip. Sampled microscope slides were sealed and placed in a microscope slide box for protection from light before observation under the Nikon_Ti fluorescence microscope.

### SEM.

Cells were grown on YCB-GlcNAc agar plates for 72 h at 25°C and then fixed with 2.5% (wt/vol) glutaraldehyde in 0.1 M sodium cacodylate buffer at 4°C overnight. Cells were then postfixed with 1% aqueous osmium tetroxide for 90 min at room temperature. Following fixation, cells were dehydrated gradually using a 15% gradient ethanol series and subsequently dried using a critical point dryer. The samples were then coated with 20 nm of gold-palladium (60:40) in an Emitech K550 sputter coater. Cells were imaged with a Hitachi S-2700 scanning electron microscope and collected with Quartz PCI software.

### Mating assays.

Cells were streaked on YCB-GlcNAc agar medium (with phloxine B) for 5 days at RT to select opaque colonies. Opaque cells of strains 3315α and 3745**a** were used as the tester strains for mating. Opaque colonies of the *MTL***a**/α *ofr1* strain were restreaked as straight lines on separate YPD and YCB-GlcNAc agar plates as the experimental strain. Opaque cells of tester strains were streaked as straight lines on YPD plates. The two sets of tester and experimental streaks were patched onto the same YPD and YCB-GlcNAc agar plates separately after 48 h of incubation at room temperature (RT). After 24 h of incubation on YPD plates and 48-h incubation on YCB-GlcNAc plates at RT, cells were replicated onto YCB-glucose selection medium lacking leucine, uridine, tryptophan, and lysine for prototrophic selection. All the plates were incubated at 30°C for 3 days before scanning and restreaking on the selection medium for further confirmation of stable prototrophic colonies (46).

Quantitative mating assays were done in liquid YCB-GlcNAc medium. Opaque cells of strains 3315α and 3745**a** were used as the tester strains for mating; SN148 **a**/αcells and SN148 **a** opaque cells were used as negative and positive controls. Opaque cells of 3315α, 3745**a**, SN148 **a,** and *ofr1* **a**/α strains were selected from YCB-GlcNAc agar medium (with phloxine B) after 5 days of culture at RT. Cells were precultured separately in liquid YCB-GlcNAc medium at RT with shaking at 220 rpm for 24 h. Then, cells were counted with a hemocytometer at ×400 magnification using an optical microscope. Cells were then centrifuged, and tester and experimental strains were mixed in 5 ml fresh YCB-GlcNAc liquid medium in 50-ml Falcon tubes at a final concentration of each strain of 1 × 10^7^ cells/ml. Cells were incubated at RT with shaking at 220 rpm for 48 h before being plated onto YCB-glucose medium for prototrophic selection; selection plates were incubated at 30°C for 3 days before the colonies were counted (45). The mating frequency is calculated based on the number of prototrophic mating product colonies (on the Trp^−^ Lys^−^ Arg^−^ plates) divided by the limiting value for the input of tester cells (detected on the Arg^−^ plates) or the experimental strain cells (detected on the Trp^−^ Lys^−^ plates). The latter two values were always similar.

### Pheromone response assays.

Approximately 5 × 10^6^ opaque cells of the *cpp1Δ/Δ MTL***a**/**a** strain were evenly streaked onto YCB-GlcNAc agar medium. SN148 (*MTL***a**/α) was used as the negative control. Single colonies of SN148 and *ofr1* **a**/α opaque cells from agar plates were selected and mixed well with 20 µl MilliQ sterile water separately. Five microliters was used for spotting onto the hyperresponsive cell streaks. The plate was incubated at 25°C for 48 h before scanning (47).

### Microarray data accession number.

The microarray data are available in the Gene Expression Omnibus (GEO) with accession number GSE75780.

## SUPPLEMENTAL MATERIAL

Figure S1  Test of opaque-specific antibodies. Confirmation of the opaque cell specificity on live *Candida* cells was done by immunofluorescence. F223-5E1-1 and F223-5H1-1 are two different monoclonal antibodies (MAbs) used in this study to identify opaque cells. Negative controls are anti-green fluorescent protein (anti-GFP) (exposure, 100 ms). Positive controls are fusion serum (opaque and hyphae) or Rb anti-*Candida* antibody (yeast) (exposures from 5 to 25 ms). Opaque-specific MAbs 5E1 and 5H1 (exposure, 25 to 50 ms on opaque cells). Secondary antibody, F(ab′)_2_ rhodamine red-X goat anti-mouse or anti-rabbit. Download Figure S1, TIF file, 0.5 MB

Figure S2  Pheromone response assays. (A) *MTL***a**/α *ofr1* opaque cells can cause sensitive *MTL***a** opaque cells to arrest. The picture shows that the cells form shmoos in the zone of inhibition under an optical microscope at ×400 magnification. (B) *MTL***a**/α *ofr1* opaque cells can respond to α pheromone. The differential interference contrast (DIC) image of shmoo was taken after 24-h pheromone treatment in liquid GlcNAc medium at ×630 magnification. Download Figure S2, TIF file, 0.7 MB

Figure S3  Sequencing result of homothallic mating product between *ofr1* mutants with *MTL***a**1 polymorphisms**.** The *ofr1* null mutant and the *ofr1* mutant from the GRACE 1.0 library with polymorphism modification on *MTL***a** were used as the parent strains. Partial **a**1 sequences from both parents are shown in black letters with highlighted polymorphisms. The mating assay was performed as described elsewhere. The auxotrophic mating products were cultured for DNA extraction. The genome DNA of the mating products was extracted, followed by a PCR to amplify the *MTL***a**1 fragment. *MTL***a**1 fragments from both parents and the mating products were sequenced. The sequencing result of homothallic mating products was shown by Chromos software. Each morphism had two peaks showing a combination of the two parents. Download Figure S3, TIF file, 0.2 MB

Figure S4  GO enrichment processes of transcription profiles between *ofr1* mutant and wild-type white cells. (A) Upregulated processes of *ofr1* mutant on glucose medium. (B) Downregulated processes of *ofr1* mutant on glucose medium. (C) Upregulated processes of *ofr1* mutant on GlcNAc medium. (D) Downregulated processes of *ofr1* mutant on GlcNAc medium. Download Figure S4, TIF file, 0.8 MB

Figure S5  Comparison of transcription profiles between the *ofr1* **a**/α opaque and white states. Both white and opaque cells were grown in glucose medium at 25°C for 12 h before total RNA extraction, followed by mRNA purification, cDNA production and labeling, and hybridization to custom microarrays as described. Left, genes significantly up- and downregulated in *ofr1* opaque states. Right, corresponding data from the literature for classical wild-type *MTL* homozygous opaque cells compared with white cells. Log_2_, >1, or log_2_, <−1; *P* value of < 0.1. The heat map figure is generated by the software MeV 4.9 MultiExperiment Viewer. Download Figure S5, TIF file, 1.1 MB

Table S1  Significantly expressed genes between *ofr1* mutant and wild-type white cells. White cells were grown at 25°C for 12 h before total RNA extraction, followed by mRNA purification and cDNA production and labeling prior to hybridization to custom microarrays as described. The results are from 2 or 3 replications, with dye swaps. Statistical significance was computed by software MeV 4.9 MultiExperiment Viewer with a *P* value of <0.1 and a cutoff log_2_ of >1 or log_2_ of <−1. Table S1, XLSX file, 0.04 MB

Table S2  Significantly expressed genes between *ofr1* mutant opaque and white cells. Both white and opaque cells were grown at 25°C for 12 h before total RNA extraction, followed by mRNA purification, cDNA production and labeling, and hybridization to custom microarrays as described. The results are from 2 replications, with dye swaps. Statistical significance was computed by software MeV 4.9 MultiExperiment Viewer with a *P* value of <0.1 and a cutoff log_2_ of >1 or log_2_ of <−1. Table S2, XLSX file, 0.04 MB

Table S3  Strains used in this study. Table S3, DOCX file, 0.01 MB

Table S4  Primers used in this study. In this study, a pair of long primers, OFR1_F and OFR1_R, were used to amplify markers *HIS1* and *URA3* by PCR from plasmids pFA-CaHIS1 and pFA-CaURA3, which provided homology to the flanking regions of *OFR1.* SN148 was used as the background strain to knock out the *OFR1* gene. Four pairs of short primers, OFR1_ex_F and OFR1_ex_R, OFR1_in_F and OFR1_in_R, HIS1-F and HIS1-R, and URA3-F and URA3-R, were used for PCR to confirm the knockouts. Table S4, DOCX file, 0.01 MB
