# Supplementary figures and images for: Deletion of a Yci1 Domain Protein of Candida albicans Allows Homothallic Mating in MTL Heterozygous Cells
Source: mBio. 2016 Apr 26;7(2):e00465-16. doi: 10.1128/mBio.00465-16 (PMC4850264; doi:10.1128/mBio.00465-16)

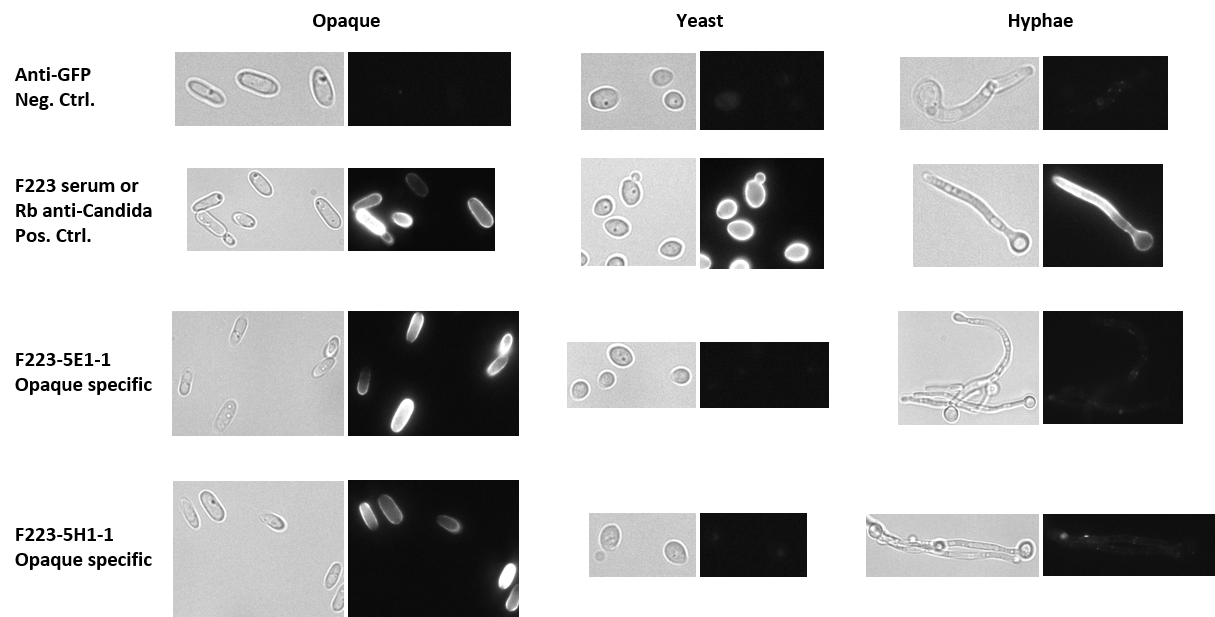

Supplement: Figure S1 — Test of opaque-specific antibodies. Confirmation of the opaque cell specificity on live Candida cells was done by immunofluorescence. F223-5E1-1 and F223-5H1-1 are two different monoclonal antibodies (MAbs) used in this study to identify opaque cells. Negative controls are anti-green fluorescent protein (anti-GFP) (exposure, 100 ms). Positive controls are fusion serum (opaque and hyphae) or Rb anti-Candida antibody (yeast) (exposures from 5 to 25 ms). Opaque-specific MAbs 5E1 and 5H1 (exposure, 25 to 50 ms on opaque cells). Secondary antibody, F(ab′)2 rhodamine red-X goat anti-mouse or anti-rabbit. Download [file mbo002162787sf1.tif]

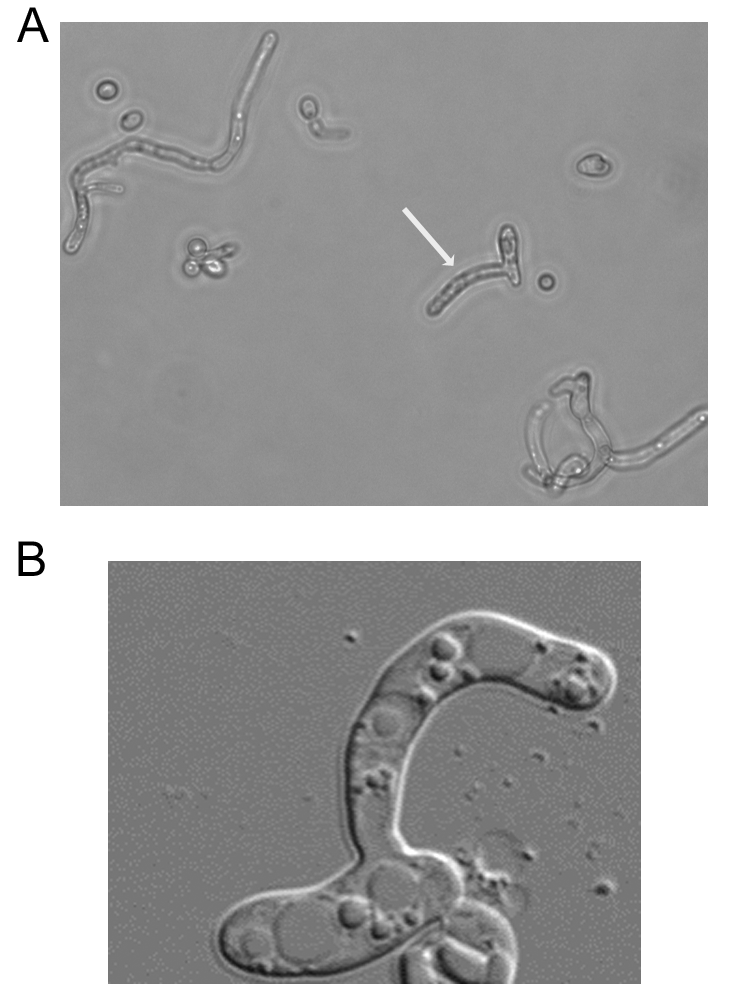

Supplement: Figure S2 — Pheromone response assays. (A) MTLa/α ofr1 opaque cells can cause sensitive MTLa opaque cells to arrest. The picture shows that the cells form shmoos in the zone of inhibition under an optical microscope at ×400 magnification. (B) MTLa/α ofr1 opaque cells can respond to α pheromone. The differential interference contrast (DIC) image of shmoo was taken after 24-h pheromone treatment in liquid GlcNAc medium at ×630 magnification. Download [file mbo002162787sf2.tif]

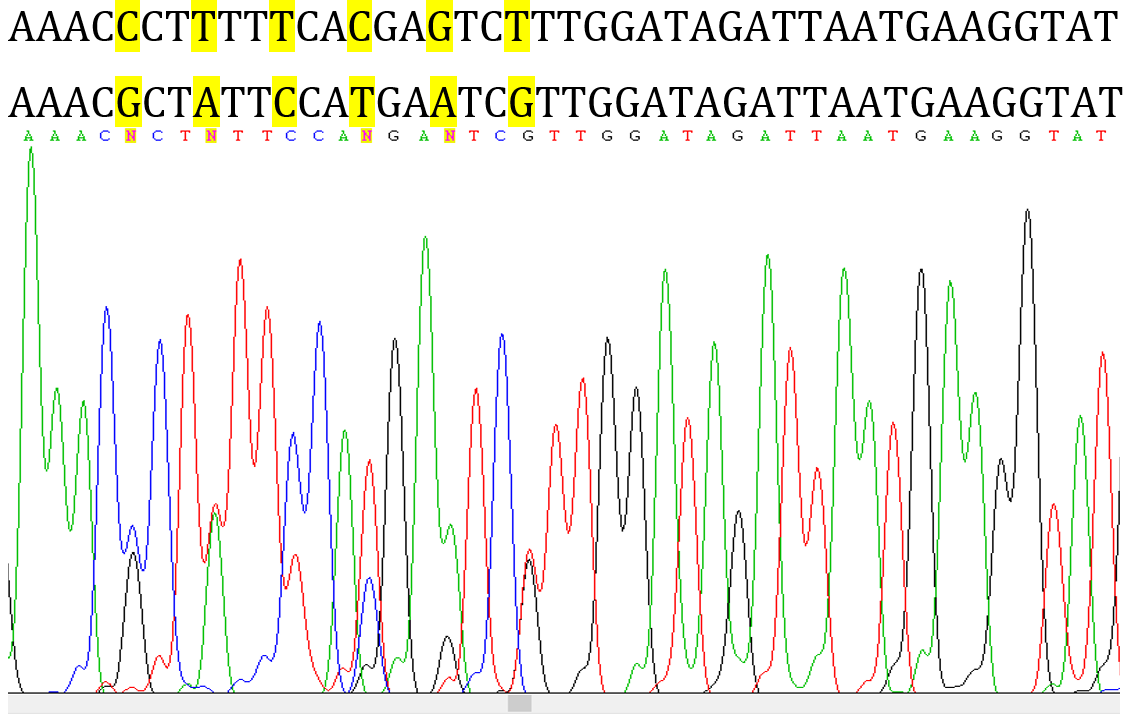

Supplement: Figure S3 — Sequencing result of homothallic mating product between ofr1 mutants with MTLa1 polymorphisms. The ofr1 null mutant and the ofr1 mutant from the GRACE 1.0 library with polymorphism modification on MTLa were used as the parent strains. Partial a1 sequences from both parents are shown in black letters with highlighted polymorphisms. The mating assay was performed as described elsewhere. The auxotrophic mating products were cultured for DNA extraction. The genome DNA of the mating products was extracted, followed by a PCR to amplify the MTLa1 fragment. MTLa1 fragments from both parents and the mating products were sequenced. The sequencing result of homothallic mating products was shown by Chromos software. Each morphism had two peaks showing a combination of the two parents. Download [file mbo002162787sf3.tif]

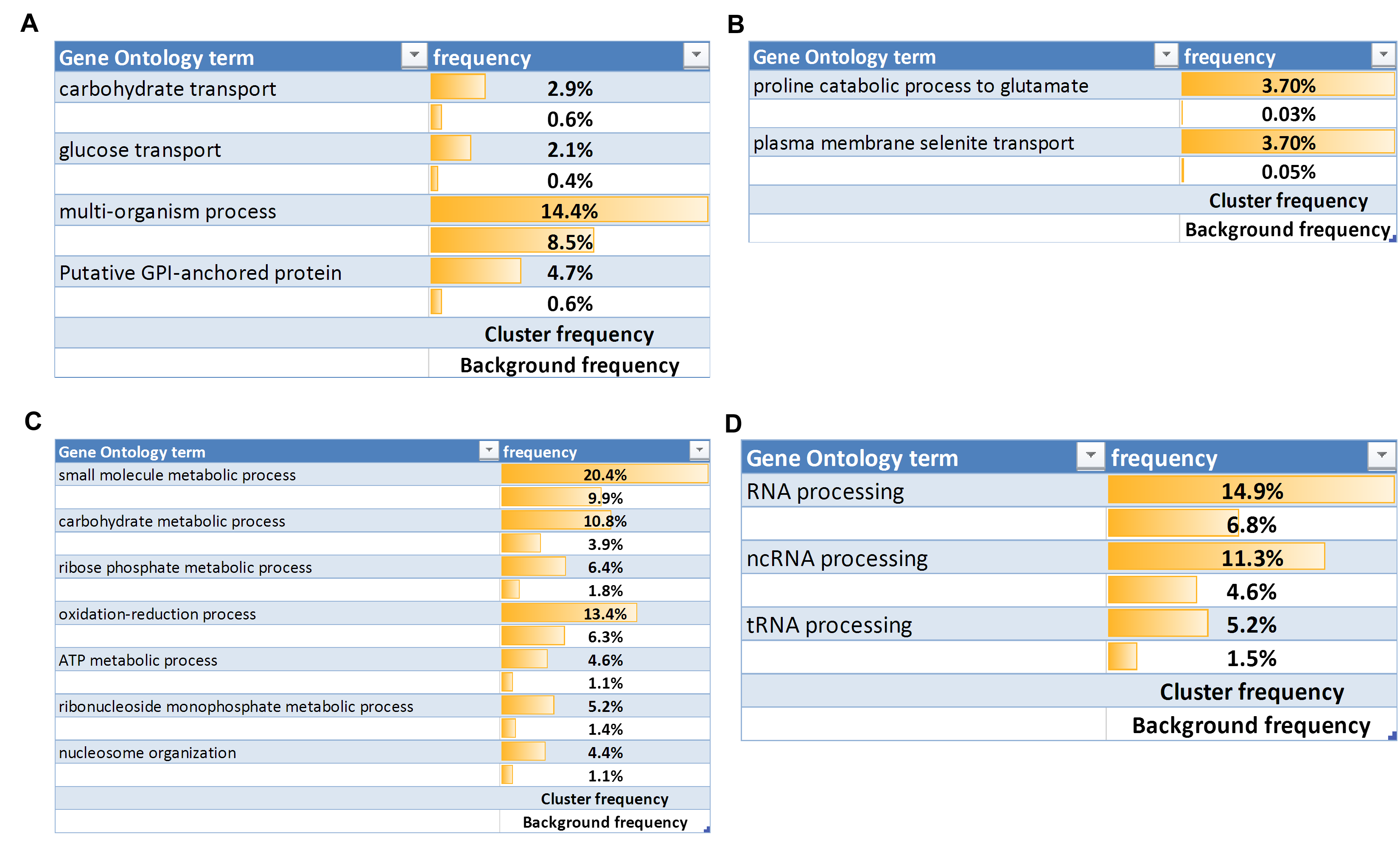

Supplement: Figure S4 — GO enrichment processes of transcription profiles between ofr1 mutant and wild-type white cells. (A) Upregulated processes of ofr1 mutant on glucose medium. (B) Downregulated processes of ofr1 mutant on glucose medium. (C) Upregulated processes of ofr1 mutant on GlcNAc medium. (D) Downregulated processes of ofr1 mutant on GlcNAc medium. Download [file mbo002162787sf4.tif]

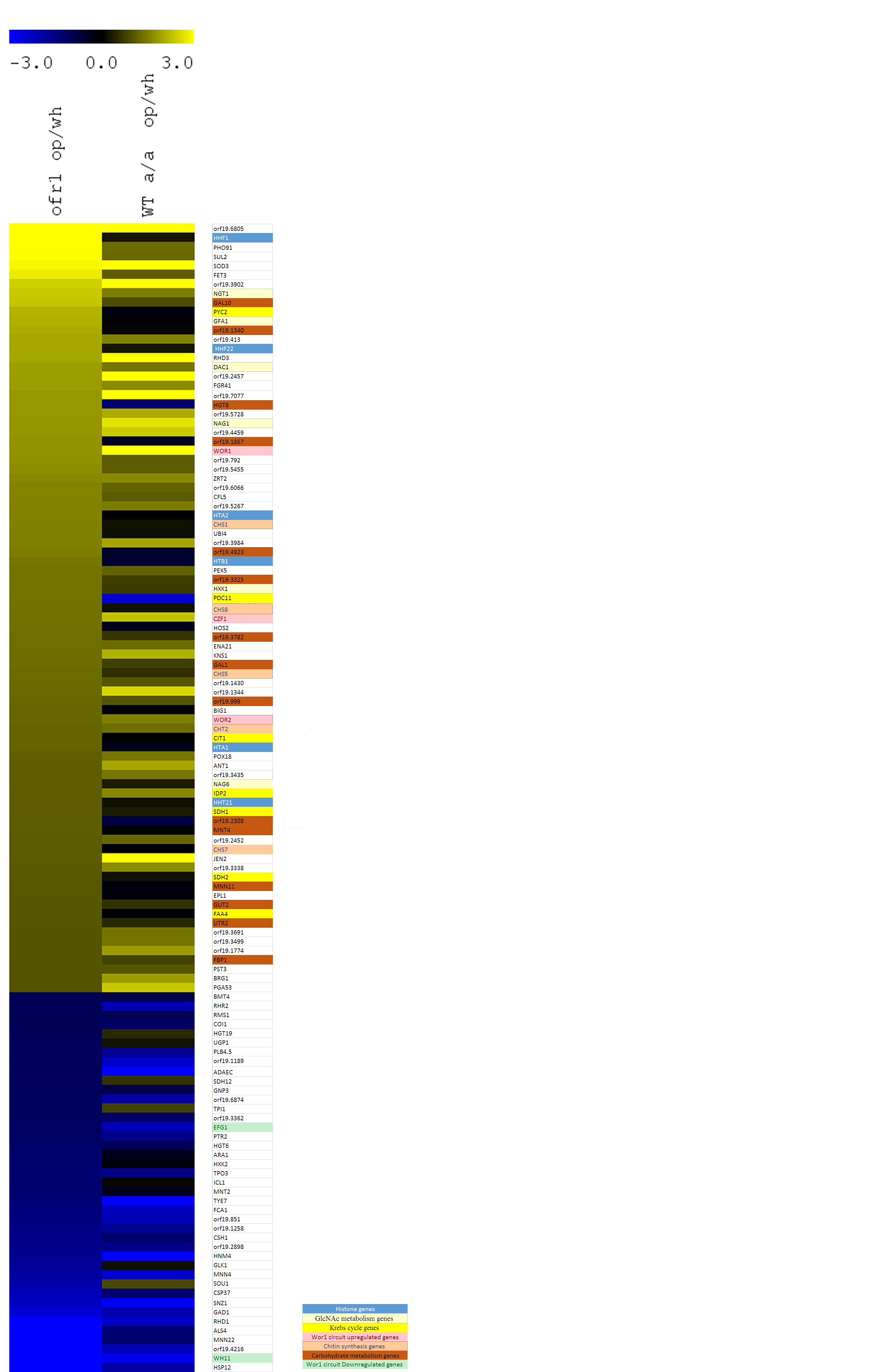

Supplement: Figure S5 — Comparison of transcription profiles between the ofr1 a/α opaque and white states. Both white and opaque cells were grown in glucose medium at 25°C for 12 h before total RNA extraction, followed by mRNA purification, cDNA production and labeling, and hybridization to custom microarrays as described. Left, genes significantly up- and downregulated in ofr1 opaque states. Right, corresponding data from the literature for classical wild-type MTL homozygous opaque cells compared with white cells. Log2, >1, or log2, <−1; P value of < 0.1. The heat map figure is generated by the software MeV 4.9 MultiExperiment Viewer. Download [file mbo002162787sf5.tif]
