# Supplementary material for: Deletion of a Yci1 Domain Protein of Candida albicans Allows Homothallic Mating in MTL Heterozygous Cells
Source: mBio. 2016 Apr 26;7(2):e00465-16. doi: 10.1128/mBio.00465-16 (PMC4850264; doi:10.1128/mBio.00465-16)
Supplement: Table S3 — Strains used in this study. [file mbo002162787st3.docx]

**Table S3**

| **Strain** | **Parent** | **Mating type** | **Description** | **Source** |
| --- | --- | --- | --- | --- |
| **SN148** | SN76 | **a**/*α* | *arg4/arg4; leu2/leu2; his1/his1; ura3 imm434/ura3 imm434; iro1 imm434/iro1 imm434* | Noble/ Johnson |
| **SN148a** | SN148 | **a/a** | *arg4/arg4; leu2/leu2; his1/his1; ura3 imm434/ura3 imm434; iro1 imm434/iro1 imm434* | Renjie Tang |
| **GRACE**  **version 1.0 library** | GRACE  library | **a**/*α* |  | Whiteway |
| **GRACE**  **library** | CASS1 | **a**/*α* |  | Merck |
| **3315** | A505 | *α /α* | *trp1/trp1; lys2/lys2* | Magee |
| **3745** | A505 | **a/a** | *trp1/trp1; lys2/lys2* | Magee |
| **CAI4 *MTL*a** | CAI-4 | **a/a** | *ura3 ::imm434/ ura3 ::imm434* | Doreen Harcus |
| **CAI4**  ***MTLα*** | CAI-4 | *α /α* | *ura3 ::imm434/ ura3 ::imm434* | Doreen Harcus |
| **CP29-17CK13** | CP29-1-7L4 | **a/a** | *ura3/ura3 cpp1 ::hisG/cpp1 ::hisG; CEK1/cek1 ::hisGURA3-hisG* | Csank |
| ***ys01*** | SN148 | **a**/*α* | *ofr1::HIS1/OFR1; arg4/arg4; leu2/leu2; ura3 imm434/ura3 imm434; iro1 imm434/iro1 imm434* | This study |
| ***ys02*** | *ys01* | **a**/*α* | *ofr1::HIS1/ofr1::URA3; arg4/arg4; leu2/leu2; iro1 imm434/iro1 imm434* | This study |
| ***ys04*** | *ys02* | **a/a** | *ofr1::HIS1/ofr1::URA3; arg4/arg4; leu2/leu2; iro1 imm434/iro1 imm434* | This study |
| ***ys05*** | *ys02* | *α /α* | *ofr1::HIS1/ofr1::URA3; arg4/arg4; leu2/leu2; iro1 imm434/iro1 imm434* | This study |
