# Supplementary material for: Deletion of a Yci1 Domain Protein of Candida albicans Allows Homothallic Mating in MTL Heterozygous Cells
Source: mBio. 2016 Apr 26;7(2):e00465-16. doi: 10.1128/mBio.00465-16 (PMC4850264; doi:10.1128/mBio.00465-16)
Supplement: Table S4 — Primers used in this study. In this study, a pair of long primers, OFR1_F and OFR1_R, were used to amplify markers HIS1 and URA3 by PCR from plasmids pFA-CaHIS1 and pFA-CaURA3, which provided homology to the flanking regions of OFR1. SN148 was used as the background strain to knock out the OFR1 gene. Four pairs of short primers, OFR1_ex_F and OFR1_ex_R, OFR1_in_F and OFR1_in_R, HIS1-F and HIS1-R, and URA3-F and URA3-R, were used for PCR to confirm the knockouts. [file mbo002162787st4.docx]

**Table S4**

| **Name** | **Description** | **Sequence (5′ to 3′)** | **Source** |
| --- | --- | --- | --- |
| **OFR1**  **_F** | *OFR1* deletion PCR cassette forward primer | ACAACCAGCTGAAAATTAGCATAAAGGAAAAGAAAGACAA  AAGAGGGGATTCAAATCGAACACATAATGGTTGGTATAGA  CGCAGCTAGTGCATTTGgaagcttcgtacgctgcaggtc | This study |
| **OFR1**  **_R** | *OFR1* deletion PCR cassette reverse primer | AGAGACACAATGAACAATAAGTGTGGAGAGTTTGTACAAG  CCATACAATCAGCAACTTCGGGATTTAAGAAGAATTTGCAA  CAGCAATAACACCTtctgatatcatcgatgaattcgag | This study |
| **OFR1**  **_ex_F** | *OFR1* external forward primer | AGAGATGAACAATATGAGAG | This study |
| **OFR1**  **_ex_R** | *OFR1* external reverse primer | TGGTGACCACGTTTGACAG | This study |
| **OFR1**  **_in_F** | *OFR1* internal forward primer | TGACTTTACGATCATTGAGG | This study |
| **OFR1**  **_in_R** | *OFR1* internal reverse primer | TAGATTCGTCAACACCATCC | This study |
| **HIS1-**  **F** | *HIS1* forward primer | TTTAGTCAATCATTTACCAGACCG | This study |
| **HIS1R** | *HIS1* reverse primer | TCTATGGCCTTTAACCCAGCTG | This study |
| **URA3**  **-F** | *URA3* forward primer | TTGAAGGATTAAAACAGGGAGC | This study |
| **URA3**  **-R** | *URA3* reverse primer | ATACCTTTTACCTTCAATATCTGG | This study |
| **MTLa**  **F** | *MTL***a**1 forward primer | TTGAAGCGTGAGAGGCAGGAG | Magee |
| **MTLa**  **R** | *MTL***a**1 reverse primer | GTTTGGGTTCCTTCTTTCTCATTC | Magee |
| **MTLα F** | *MTLα*2 forward primer | TTCGAGTACATTCTGGTCGC | Magee |
| **MTLα R** | *MTLα*2 reverse primer | TGTAAACATCCTCAATTGTACCCG | Magee |
